# Osteopontin Promotes Expression of Matrix Metalloproteinase 13 through NF-*κ*B Signaling in Osteoarthritis

**DOI:** 10.1155/2016/6345656

**Published:** 2016-08-30

**Authors:** Yusheng Li, Wei Jiang, Hua Wang, Zhenhan Deng, Chao Zeng, Min Tu, Liangjun Li, Wenfeng Xiao, Shuguang Gao, Wei Luo, Guanghua Lei

**Affiliations:** ^1^Department of Orthopaedics, Xiangya Hospital, Central South University, Changsha 410078, China; ^2^Department of Bone and Joint, The Second Clinical Medical College (Shenzhen People's Hospital), Jinan University, Shenzhen 518020, China; ^3^Department of Orthopaedics, Second People's Hospital of Jingmen, Jingmen 448000, China; ^4^Department of Joint Surgery, Changsha Central Hospital, Changsha 410000, China

## Abstract

Osteopontin (OPN) is associated with the severity and progression of osteoarthritis (OA); however, the mechanism of OPN in the pathogenesis of OA is unknown. In this study, we found that OA patients had higher abundance of OPN and matrix metalloproteinase 13 (MMP13). In chondrocytes, we showed that OPN promoted the production of MMP13 and activation of NF-*κ*B pathway by increasing the abundance of p65 and phosphorylated p65 and translocation of p65 protein from cytoplasm to nucleus. Notably, inhibition of NF-*κ*B pathway by inhibitor suppressed the production of MMP13 induced by OPN treatment. In conclusion, OPN induces production of MMP13 through activation of NF-*κ*B pathway.

## 1. Introduction

Osteoarthritis (OA) is regarded as the most prevalent chronic joint disease. OA is characterized by a group of mechanical abnormalities including degradation of articular cartilage, thickening of subchondral bone, synovial inflammation, and formation of osteophytes, leading to chronic pain and functional restrictions in affected joints, substantial morbidity, physical disability, and reduced quality of life [1–3]. Data from the World Health Organization (WHO) estimated that about 10% of men and 18% of women, as well as 65% of all those with age more than 60 years, have symptomatic OA [4]. Although the etiology of OA is complex, including reasons from the genetic, constitutional, and biomechanical aspects, we have growing knowledge and understanding on the pathogenesis of OA [3].

OPN (osteopontin) is a 44~75 KD multifunctional phosphoprotein and is known as early T cell activation gene-1 (Eta-1) [5, 6]. OPN is secreted by many types of cells, including macrophages, lymphocytes, epithelial cells, vascular smooth muscle cells, and even chondrocytes as well as synoviocytes [7–10]. OPN is highly abundant in the extracellular fluids at sites of inflammation, extracellular matrix of mineralised tissues, and even in the bone [7, 9, 11]. In the bone, OPN regulates the cell-matrix and cell-cell interaction, the cartilage-to-bone transition in fracture repair, and the attachment of osteoclasts to the bone matrix [6, 12, 13]. Interestingly, mRNA expression and protein abundance of OPN are associated with the pathogenesis of OA. At the beginning, a study found that mRNA expression of OPN isolated from human OA cartilage is higher compared to the normal cartilage [14]. Subsequently, increased abundance of OPN in the plasma, synovial fluid, and articular cartilage in OA patients were found [3, 15, 16], indicating that expression of OPN is associated with progressive joint damage and the severity and progression of OA.

The most remarkable biochemical change in OA is the progressive loss of articular cartilage, which contains two main extracellular matrix macromolecules: type II collagen and aggrecan, a major component of the cartilage-specific proteoglycans [17–19]. The matrix metalloproteinases (MMPs), including collagenases (MMP1 and MMP13), gelatinases (MMP2 and MMP9), and stromelysin (MMP3), mediate cartilage collagen breakdown, whereas aggrecanases, which are members of the A disintegrin and metalloproteinase with thrombospondin motifs (ADAMTS) family including ADAMTS1, ADAMTS4, ADAMTS5, ADAMTS8, ADAMTS9, and ADAMTS15, mediate loss of cartilage aggrecan. Thus, the importance of MMPs and ADAMTS in the pathogenesis and development of OA is widely demonstrated [20–23]. Intriguingly, recent investigations have shown that OPN affects the expression of MMP13 [17, 24]. However, the mechanism by which OPN regulates expression of MMP13 still remains to be known. NF-*κ*B pathway has critical roles in the expression of inflammatory factors, including MMP13 [25–28]. Thus, the hypothesis of this study is that OPN regulates expression of MMP13 through NF-*κ*B pathway.

## 2. Materials and Methods

### 2.1. Cartilage Acquisition and Assessment

The study was approved by the institutional review board and ethics committee of Xiangya Hospital affiliated to Central South University, which conformed with the regulations of medical ethics. An informed consent about this experiment was obtained from all subjects. A written informed consent to participate in this study was provided by participants. The normal cartilage tissues from non-OA patients and degenerated cartilage tissues from OA patients were obtained in previous studies [15, 17, 29, 30]. The cartilage tissues were assessed with hematoxylin-eosin (HE) and safranin-O staining and a modified Mankin grading system in previous studies [15, 17, 29, 30].

### 2.2. Cell Isolation and Culture Conditions

The chondrocytes were isolated and cultured according to previous studies [17, 29, 30]. Briefly, samples were minced into pieces of less than 1 mm^3^, followed by sequential digestion at 37°C with 0.15% collagenase II (Invitrogen, Carlsbad, CA, USA) for 5-6 h with stirring every 20 min after 2 h. Chondrocytes were isolated after centrifugation and cultured in DMEM-F12 containing 10% fetal bovine serum (FBS) and antibiotics for 5–7 days before use. For OPN treatment, OPN (recombinant human osteopontin, R&D Systems, Minneapolis, MN, USA) was added to the medium with the dosage of 0, 0.5, 1, 2, and 4 *μ*g/mL for 24, 48, or 72 hours. For OPN inhibition, siRNA targeting OPN was transiently transfected into cells using Lipofectamine*™* 2000 reagent (Invitrogen Life Technologies, San Diego, CA, USA) according to previous studies [29, 31]. siRNA sequences were OPN-siRNA, with sequence as 5′-CCU GUG CCA UAC CAG UUA ATT-3′ and antisense 5′-UUA ACU GGU AUG GCA CAG GTT-3′ [29]. For p65 protein translocation inhibition, pyrrolidinedithiocarbamic (PDTC) acid was used with 100 *μ*M.

### 2.3. RT-PCR

RT-PCR analysis was performed according to previous reports [25]. Briefly, total RNA was isolated from liquid nitrogen frozen samples using TRIzol regent (Invitrogen, USA) and then treated with DNase I (Invitrogen, USA) according to the manufacturer's instructions. Synthesis of the first strand (cDNA) was performed using oligo (dT) 20 and SuperScript II Reverse Transcriptase (Invitrogen, USA). Primers used in this study were designed with Primer 5.0. Sequences of all primers used were as follows: MMP13-F: 5′-CTTAGAGGTGACTGGCAA AC-3′; MMP13-R: 5′-GCCCATCAAATGGGTA GAA G-3′; OPN-F: 5′-GTGGGA AGG ACA GTT ATG AA-3′; OPN-R: 5′-CTG ACT TTG GAA AGT TCC TG-3′; GAPDH-F: 5′-TGA CTT CAA CAG CGA CAC CCA-3′; and GAPDH-R: 5′-CAC CCT GTT GCT GTA GCC AAA-3′. GAPDH was used as an internal control to normalize target gene transcript levels.

### 2.4. Immunoblotting

Western blot analysis was conducted according to previous study [25]. Briefly, SDS-PAGE is used to separate protein obtained from samples. Then, the separated protein is transferred to PVDF membranes (Millipore, MA, USA) and incubated with primary antibodies overnight at 4°C after the blockage with 5% nonfat milk in Tris-Tween buffered saline buffer (20 mM Tris, pH 7.5, 150 mM NaCl, and 0.1% Tween-20) for 3 h. Following is the information for antibodies: anti-OPN (AP11567a, Abgent, CA, USA), MMP13 (ALS10395, Abgent, CA, USA), anti-p65 (BS4135, Bioworld, MN, USA), anti-p-p65 (#3031, CST, USA), and anti-COL2A1 (sc-28887, Santa Cruz Biotechnology, Texas, USA). Further, HRP-conjugated secondary antibodies were incubated for 1 h at room temperature before analysis the signal intensity using AlphaImager 2200 software (Alpha Innotech Corporation, CA, USA).

### 2.5. Immunofluorescence

Immunofluorescence staining was performed at room temperature according to previous studies [32, 33]. Firstly, the cells were fixed by 4% paraformaldehyde (PFA, Sigma-Aldrich) and then permeabilized with 0.5% Triton X-100 (Sigma-Aldrich) solution in phosphate buffered saline (PBS) and incubated with blocking solution (3% bovine serum albumin (BSA, Sigma-Aldrich) diluted in 0.1% Triton X-100). Then, before washing with PBS, cells were incubated firstly with polyclonal rabbit antibodies against anti-NF-*κ*B p65 (BS4135, Bioworld, MN, USA) for 24 h (1 : 250) and then with FITC-conjugated goat anti-rabbit IgG for 1 h. Further, coverslips were mounted with GEL/MOUNT (Biomeda, Foster City, CA, USA) and then visualized the fluorescence under a confocal fluorescence microscope (Carl Zeiss, Göttingen, Germany).

### 2.6. Statistical Analyses

Data shown are the means ± the standard error of the mean (SEM). All statistical analyses for data were performed using SPSS 16.0 software (Chicago, IL, USA). Data were analyzed between two groups using Student's* t*-test, while among more than two groups data were analyzed by the one-way ANOVA method. Differences of *P* < 0.05 were considered significant.

## 3. Results

### 3.1. OPN Promotes Expression of MMP13

**Figure 1:**
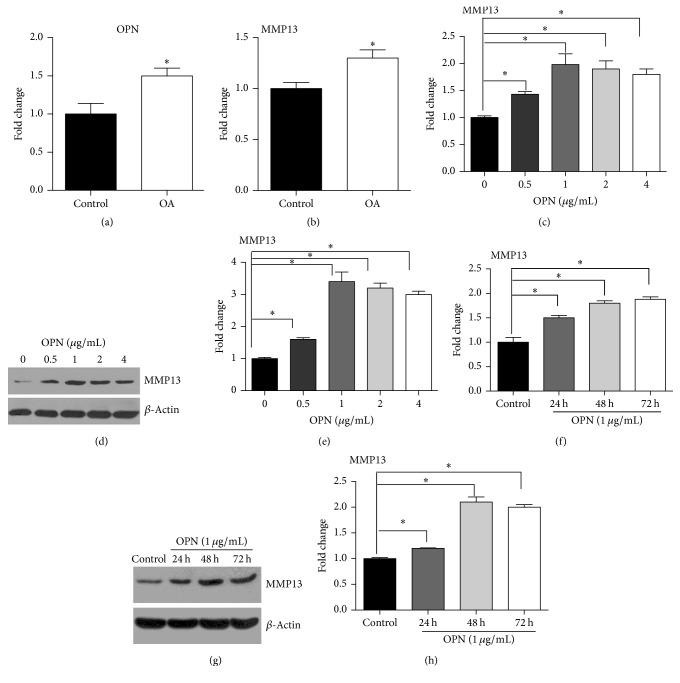
OPN promotes expression of MMP13. ((a)-(b)) The expression of OPN ((a), *n* = 13) and MMP13 ((b), *n* = 13) in normal cartilage tissues obtained from non-OA patients and degenerated cartilage tissues from OA patients. ((c)–(e)) OPN treatment at indicated concentrations promotes expression of MMP13 at mRNA (c) and protein levels ((d) and (e)) in the chondrocytes. (d) Immunoblotting of MMP13. (e) Quantification of relative MMP13 abundance from data shown in (d). ((f)–(h)) OPN treatment at indicated time promotes expression of MMP13 at mRNA (f) and protein levels ((g) and (h)) in the chondrocytes. (g) Immunoblotting of MMP13. (h) Quantification of relative MMP13 abundance from data shown in (g). Data are representative of two independent experiments with 4–6 repeats per group. *∗* indicates a statistically significant difference between two groups (*P* < 0.05). OA: osteoarthritis; OPN: osteopontin; and MMP13: matrix metalloproteinase 13.

Expressions of OPN and MMP13 were evaluated in normal cartilage tissues obtained from non-OA patients and degenerated cartilage tissues from OA patients. The cartilage tissues from OA patients have significantly (*P* < 0.05) higher expression of OPN compared to the normal cartilage tissues (Figure 1(a)). Also, expression of MMP13 significantly (*P* < 0.05) increased in the cartilage tissues from OA patients, compared to the normal cartilage tissues (Figure 1(b)). To characterize the cause and effect relationship between OPN and MMP13, we treated the chondrocytes with different dosage of OPN. As indicated in Figure 1(c), OPN treatment significantly (*P* < 0.05) induced mRNA expression of MMP13 in chondrocytes. Indeed, similar result was found in protein abundance of MMP13 in chondrocytes (Figures 1(d) and 1(e), *P* < 0.05). Also, we analyzed expression of MMP13 in chondrocytes, treated with OPN at the dosage of 1 *μ*g/mL. OPN supplementation remarkably promoted mRNA expression of MMP13 at 24, 48, and 72 hours after supplementation (Figure 1(f), *P* < 0.05). Similarly, we also observed that OPN supplementation significantly increased protein abundance of MMP13 in chondrocytes at 24, 48, and 72 hours after supplementation (Figures 1(g) and 1(h), *P* < 0.05). Collectively, expression of OPN increases in cartilage tissues from OA patients, and OPN promotes expression of MMP13.

### 3.2. OPN Promotes Expression of MMP13 through NF-Kappa B Signaling

**Figure 2:**
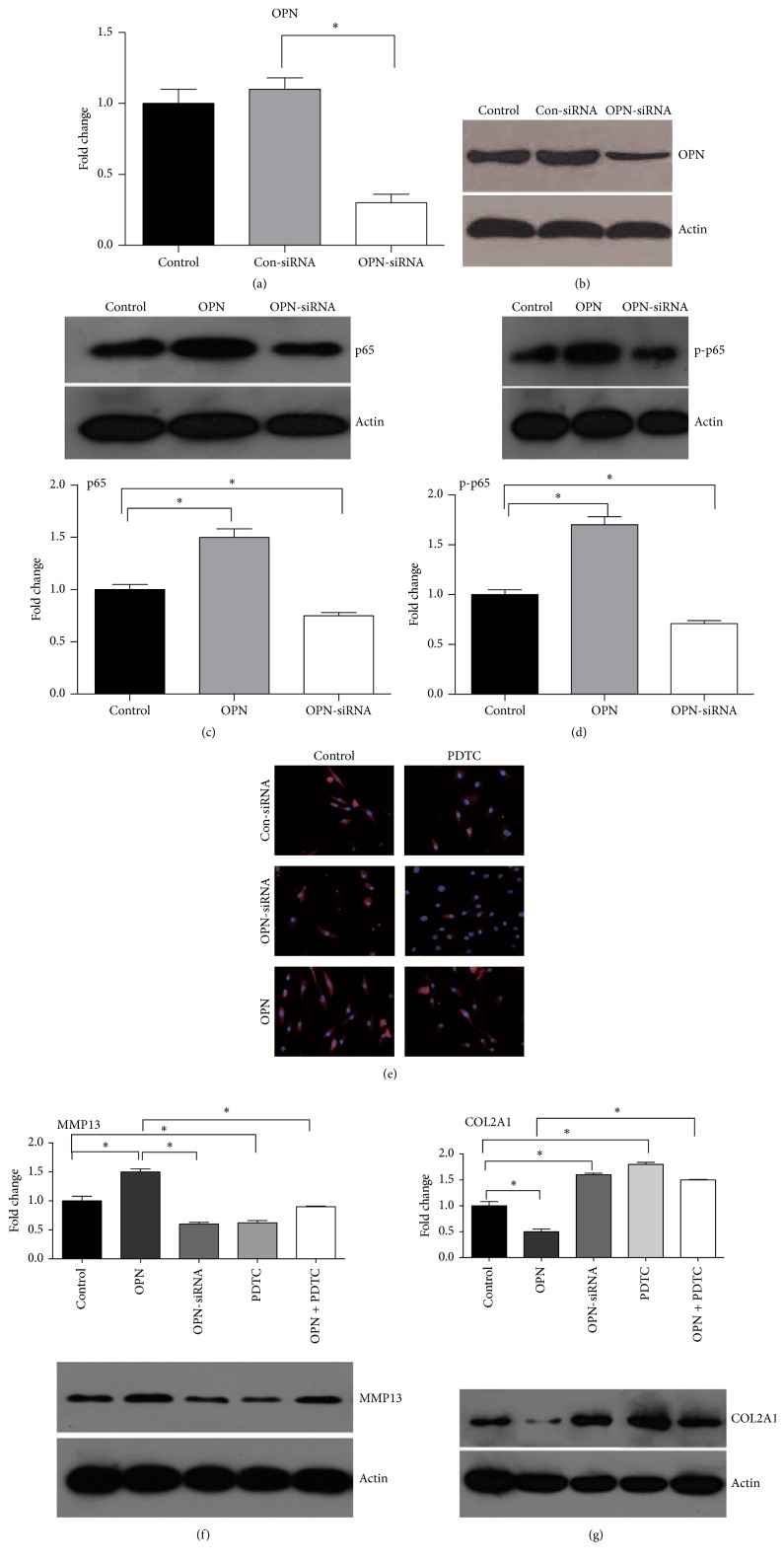
OPN promotes expression of MMP13 through NF-kappa B signaling. ((a)-(b)) The expression of OPN after OPN targeting siRNA in chondrocytes from mRNA expression (a) and protein levels (b). ((c)-(d)) The abundance of p65 (c) and phosphorylated p65 (d) after OPN treatment or OPN-siRNA treatment. (c) Immunoblotting of p65 (upper part) and quantification of relative p65 abundance (bottom) from data shown in upper part. (d) Immunoblotting of p-p65 (upper part) and quantification of relative p-p65 abundance (bottom) from data shown in upper part. (e) The translocation of p65 protein from the cytoplasm to the nucleus in indicated situations (×100). ((f)-(g)) The protein abundance of MMP13 (f) and COL2A1 (g) in indicated situations. (f) Immunoblotting of MMP13 (bottom) and quantification of relative p65 abundance (upper part) from data shown in bottom. (g) Immunoblotting of COL2A1 (bottom) and quantification of relative p65 abundance (upper part) from data shown in bottom. Data are representative of two independent experiments with 4–6 repeats per group. *∗* indicates a statistically significant difference between two groups (*P* < 0.05). OPN: osteopontin and MMP13: matrix metalloproteinase 13.

To characterize the mechanism by which OPN regulates expression of MMP13, we focused on the NF-kappa B pathway in chondrocytes because this signaling has a pivotal role in the expression of inflammatory factors after stimulation [25, 26, 34]. We transfected the chondrocytes with OPN targeting siRNA, which significantly (*P* < 0.05) lowered mRNA expression and protein abundance of OPN in chondrocytes (Figures 2(a) and 2(b)). As the control, control siRNA has little effect on mRNA expression and protein abundance of OPN in chondrocytes (Figures 2(a) and 2(b)). In the chondrocytes, OPN treatment significantly (*P* < 0.05) increased the abundance of p65 and phosphorylated p65, compared to the nontreatments (Figures 2(c) and 2(d)). However, the abundance of p65 and phosphorylated p65 in chondrocytes with OPN-siRNA treatment was significantly (*P* < 0.05) lower compared to the controls (Figures 2(c) and 2(d)). Also, we found that OPN treatment significantly promoted translocation of p65 protein from the cytoplasm to the nucleus, while OPN-siRNA treatment inhibited this translocation, compared to the controls (Figure 2(e)). As the control, PDTC treatment inhibited translocation of p65 protein from the cytoplasm to the nucleus (Figure 2(e)). In the chondrocytes, OPN treatment significantly (*P* < 0.05) increased protein abundance of MMP13, while OPN-siRNA treatment lowered (*P* < 0.05) protein abundance of MMP13 (Figure 2(f)). Although OPN treatment enhanced the protein abundance of MMP13, PDTC treatment reversed (*P* < 0.05) abundance of MMP13 caused by OPN treatment (Figure 2(f)). Indeed, PDTC along with treatment also decreased protein abundance of MMP13 in the chondrocytes (Figure 2(f)). OPN treatment significantly (*P* < 0.05) decreased protein abundance of COL2A1 in the chondrocytes, while OPN-siRNA treatment increased (*P* < 0.05) protein abundance of COL2A1 (Figure 2(e)). Although OPN treatment inhibited protein abundance of COL2A1 in the chondrocytes, PDTC treatment alleviated (*P* < 0.05) the lower abundance of COL2A1 induced by OPN treatment (Figure 2(e)). Summarily, OPN activates the NF-kappa B pathway, which in turn promotes expression of MMP13.

## 4. Discussion

Although most investigations have shown that increased OPN is associated with the progressive joint damage and the disease severity and progression of OA [15, 35], some reports have a contradictory conclusion. For example, one study has found that OA patients have lower concentration of OPN compared to the healthy controls [36]. Also, with OPN-deficient mice, OPN deficiency results in aging-associated and instability-induced OA [24], suggesting that it is required for cartilage homeostasis and preventing OA progression. These compelling findings are suggesting that OPN has complex roles in joint homeostasis and in the pathogenesis of OA. Indeed, this is supported by our discovery that OPN could regulate the expression of various factors associated with the pathogenesis of OA, including hypoxia-inducible factor-2*α* [29], ADAMTS4 [30], tissue inhibitors of metalloproteinases [37], interleukin-6 and interleukin-8 [38], and even caveolin-1 [39]. Similar to the previous study, we found that mRNA expression of OPN increases in the cartilage tissues from OA patients. Also, increased mRNA expression of MMP13 was found in the cartilage tissues from OA patients. This leads to the hypothesis that increased OPN promotes expression of MMP13. Indeed, in the chondrocytes, we found that OPN increases mRNA expression and protein abundance of MMP13. Similarly, a previous study has found that both OPN and phosphorylated OPN promote expression of MMP13 at both mRNA and protein levels [17].

NF-*κ*B pathway has critical roles in the expression of inflammatory factors [25, 26]. In unstimulated situation, the NF-*κ*B dimers are sequestered in the cytoplasm by the inhibitor of *κ*Bs (I*κ*B), which keeps NF-*κ*B proteins in the cytoplasm by masking the nuclear localization signals of NF-*κ*B proteins with the ankyrin repeat domains of I*κ*B [34]. Upon activation, I*κ*B was phosphorylated, leading to targeting for the proteasomal degradation and to release the NF-*κ*B proteins [34]. Subsequently, the NF-*κ*B proteins are moved into the nucleus and turn on the expression of specific genes that have NK-*κ*B binding elements in their promoter or other sites [34]. Interestingly, NF-*κ*B pathway has been reported to regulate the expression of MMP13. For example, high-mobility group box chromosomal protein 1 (HMGB1) or lipopolysaccharide (LPS) significantly promotes the expression of MMP13 and the activation of NF-*κ*B pathway by increasing the I*κ*B phosphorylation, while it inhibits the activation of NF-*κ*B pathway by NF-*κ*B inhibitor (Bay 11-7085) which remarkably inhibits expression of MMP13 induced by HMGB1 and LPS [27]. CpG oligodeoxynucleotides treatment promotes the expression of MMP13 and the activation of NF-*κ*B pathway in the murine odontoblast-lineage cell line, while the treatment with inhibitors of NF-*κ*B pathway, including NF-*κ*B inhibitors (PDTC), I*κ*B*α* phosphorylation inhibitors (Bay 11-7082), or I*κ*B protease inhibitor (L-1-tosylamido-2-phenylethyl chloromethyl ketone, TPCK), markedly suppresses MMP13 expression induced by CpG ODN [28]. Notably, OPN has been shown to induce the activation of NF-*κ*B pathway and the expression of NF-*κ*B pathway-dependent factors in breast cancer cells [40]. Similarly, OPN promotes expression of MMP2 and MMP9 through NF-*κ*B pathway [41]. In line with these investigations, this study shows that OPN treatment promotes activation of NF-*κ*B pathway by increasing the abundance of p65 and phosphorylated p65 and translocation of p65 protein from the cytoplasm to the nucleus. Inhibition of expression of OPN inactivates the NF-*κ*B pathway. Importantly, the current study found that OPN increases expression of MMP13 through activation of NF-*κ*B pathway, which is similar to a previous conclusion that OPN activates NF-*κ*B pathway and promotes the expression of NF-*κ*B- dependent factors [40, 41]. However, besides the NF-*κ*B pathway, other signal pathways are also associated with expression of MMP13. For example, SB203580, the inhibitor of p38 signal pathway, significantly reduces expression of MMP13 induced by TNF-*α* treatment [42] or others [27]. Thus, it is interesting to know whether OPN also regulates MMP13 expression through other signaling pathways.

In conclusion, current study verified that expression of OPN and MMP13 is increased in OA, and OPN promotes expression of MMP13 through activation of NF-*κ*B pathway in OA. The discovery of this study has great potentials for understanding the pathogenesis of OA and treatment of OA through manipulation of NF-*κ*B pathway.
